# Trust Versus Content in Multi-functional Land Management: Assessing Soil Function Messaging in Agricultural Networks

**DOI:** 10.1007/s00267-022-01647-2

**Published:** 2022-04-22

**Authors:** Lilian O’Sullivan, Cees Leeuwis, Linde de Vries, David P. Wall, Talke Heidkroß, Kirsten Madena, Rogier P. O. Schulte

**Affiliations:** 1 Teagasc Crops, Environment and Land Use Programme, Johnstown Castle, Co. Wexford, Ireland; 2 Farming Systems Ecology Group, Wageningen University and Research, 6708B Wageningen, The Netherlands; 3 Knowledge Technology and Innovation Group, Wageningen University and Research, 6700EW Wageningen, The Netherlands; 4 Landwirtschaftskammer Niedersachsen, Oldenburg, Germany

**Keywords:** Social network analysis, Soil functions, AKIS, Sustainability, Functional land management

## Abstract

Growing sustainability demands on land have a high knowledge requirement across multiple scientific domains. Exploring networks can expose opportunities for targeting. Using mixed-methods combining social network analysis (SNA) and surveys, networks for key soil functions in case studies in Germany, Ireland and the Netherlands are explored. We find a diversity of contrasting networks that reflect local conditions, sustainability challenges and governance structure. Farmers were found to occupy a central role in the agri-environmental governance network. A comparison of the SNA and survey results indicate low acceptance of messages from many central actors indicating scope to better harness the network for sustainable land management. The source of the messages was important when it came to the implementation of farm management actions. Two pathways for enhanced farmer uptake of multi-functionality are proposed that have wider application are; to increase trust between farmers and actors that are agents of multi-functional messages and/or to increase the bundling or multi-functionality of messages (mandate) of actors trusted by farmers.

## Introduction

### Policy Context

Within the European Union (EU), the Common Agricultural Policy (CAP) is the key agri-environmental instrument and represents the largest agricultural support system in the world (Pe’er et al. 2014). The early decades of the CAP have played a critical role in shaping land management in favour of food production. By the 1980s, guaranteed commodity prices had generated an over-supply of food products and an intensification of agriculture that aligned with environmental deterioration (Pe’er et al. 2014). To address this, successive CAP reforms have expanded agricultural policy to incorporate environmental concerns and the sustainability of rural ecosystems. Although the CAP is now recognised as the largest funding source for nature conservation in Europe (Keenleyside and Tucker 2010; Herzon et al. 2018) a review of the CAP 2014–2020 period indicated that green payments for sustainable agriculture have neither met the environment and climate needs of Member States (MS) nor wider CAP objectives (Meredith and Hart 2019). In 2018, the European Commission (EC) published a legislative proposal on the CAP period 2021–2027. Key elements of this proposal include: (1) better targeting of funding towards small and medium-sized farms; (2) guaranteeing a higher ambition on environmental and climate action; (3) putting agriculture at the heart of European society and (4) making greater use of knowledge and innovation (EC 2018a, 2018b). As anticipated, the proposal makes significant contributions to the European Green Deal and the Farm to Fork and Biodiversity Strategies (EC 2020). A new delivery model based upon strategic plans (SP) that reflect MS needs, is expected to facilitate more context-specific policies (EC 2018).

### The challenge

Agricultural policy reforms increasingly link agriculture to the delivery of public goods and services. Farmers receive policy support as producers and increasingly as natural resource managers in recognition of the more diverse role of agriculture from an ecosystem perspective. An abundance of policy instruments relevant to land management at farm and national scale have emerged under environmental directives that have largely been formulated independently of each other (Schulte et al. 2015, 2019). Historically, agri-environmental sustainability has been steered by direct regulatory processes focused on mandatory operating requirements targeted towards solving environmental problems (Taylor et al. 2012). However, since the 1990s, there has been a shift from government-based to multi-actor governance due to the failure of traditional policies to address the challenges of environmental degradation and biodiversity loss (Loft et al. 2015). This multi-actor governance refers to an expansion of the decision-making process on the provision and use of public goods to a much wider range of stakeholders (Maury et al. 2013). This implies that sustainability depends also on the role of different actors. Faced with a growing suite of competing societal demands on land (Schulte et al. 2019), farmers and stakeholders have to absorb and consider increasingly complex knowledge regarding both global and local processes to achieve sustainability (Leeuwis 2004). This complexity presents particular challenges for farmers and other land managers with direct responsibility for land use and soil management decisions. The point of obligation of territorial agri-environmental policies typically applies at farm scale, meaning that farmers are assigned a key role with respect to the practical implementation of practices and measures for sustainable land management. European policies have fostered the model of family-operated farms, typically led and managed by one or two people. Within the context of increasing societal expectations, a high knowledge requirement across multiple scientific domains is now imposed on individual farmers. In the context of a network, knowledge (and information linked to other policy instruments) is communicated to farmers by means of messages that originate from different sources in the network. This can result in information overload. As the demands for sustainability are increasing, this can be associated with an implicit expansion of the amount of messaging and information from science, policy, markets and value chains that farmers receive, which may increase the potential for information overload at farm scale.

### Theoretical Framework

Functional Land Management (FLM) is an integrated approach that aims to optimise the delivery of land-based ecosystem services (Schulte et al. 2014). Five ubiquitous agricultural land-based ecosystem services, or soil functions, have been identified by Schulte et al. (2014), based on Haygarth and Ritz (2009): (1) primary production of food, feed, fuel and fibre; (2) water purification and regulation; (3) carbon storage, sequestration and climate regulation; (4) provision of habitats for biodiversity and (5) provision and cycling of nutrients. These five key soil functions represent a large portion of societal demand for land-based ecosystem services with pertinence for agri-environmental governance within the EU (Schulte et al. 2015, 2019). Accordingly, the FLM framework offers a mechanism by which networks for sustainable land management can be framed. Already an EU level assessment of the societal demand (reflected by policy indicators) for multi-functionality based upon FLM was found to differ between MS indicating that SP represent an opportunity for more effective and targeted incentivisation of sustainable land management (Schulte et al. 2019). While that work provides insights into which soil functions should be prioritised where, further questions emerge, including who and how to target messages for FLM. The goal of this work therefore is to address this knowledge gap and to explore networks for multi-functionality using the five soil functions in the FLM framework to understand whether similarities or differences exist between networks in different contexts for the same key soil functions from which high-level conclusions can be drawn.

In relation to multi-functional social networks for FLM, the following are important considerations outlined in brief below; (1) the potential of particular actors to bundle messages, (2) the coherence of messages that actors receive and (3) the role of trust and message acceptance.

The expanded governance context gives rise to potentially alternative points of entry for agri-environmental sustainability, for example through targeting different actors who communicate messages in the network. Targeting “higher” entry points could potentially address the complexity of messages through bundling. Bundling refers to messages that address multiple soil functions simultaneously. Actors that bundle could be targeted to align messages in favour of fostering sustainable land management. Actors who transmit messages related to multiple soil functions (bundle) may have a particular role to streamline messaging for multiple functions. Off-farm organisations may employ more people and so scope to bundle and align messages could be leveraged.

Coherence can be defined as a lack of contradiction or the degree of agreement between different elements in a set (Toro-Alvarez 2020). Cognitions refer to the opinions, knowledge and beliefs about oneself and their environment and when two cognitions are inconsistent this can generate dissonance (Festinger 1957). This cognitive dissonance, also called incoherence, creates a state of discomfort and can promote a search for coherence (Toro-Alvarez 2020). Within a network, messages are communicated to farmers from multiple different sources. The messages related to soil functions are likely to come from multiple sources but refer to the same resource and could therefore result in incoherence for message recipients. At an applied level, coherence has been linked to several criteria of success (see Toro-Alvarez 2020 for overview). Understanding the level of coherence that farmers associate with messages related to sustainable land management could provide insight into whether a need for greater coherence exists. In turn, this could help inform needs and mechanisms to support sustainable land management at a practical level.

According to Social Judgement Theory, the messages that people receive are compared to their current point of view, upon which different attitudes are formed that fall into different zones of acceptance (Sherif and Hovland 1961; Sherif et al. 1965; Mallard 2010). The level of involvement, or how important an issue is to the person’s life will affect where on the scale the individual will position the message received, which may fall into the latitudes of acceptance, rejection or non-commitment (Sherif and Hovland 1961). Although individuals judge messages according to their own so-called anchor or standpoint, it is important that this anchor is movable. If messages fall within the latitude of acceptance, it is perceived as closer to their viewpoint and the anchor is moved closer to the message, however if outside the latitude of acceptance, the message is perceived as further away (Griffin 2012). Acceptance is multi-faceted, and can be linked to different layers including acceptance of the underlying problem, acceptance of the solutions, credibility and trust in the change agent and acceptance of the consequences of the innovation (Leeuwis 2004). Based upon the adoption and diffusion research, Leeuwis (2004) indicate that increasing acceptance, and ultimately adoption of sustainability innovations, a number of different stages occur including awareness, interest, evaluation, trialling of the proposed innovation and adoption/acceptance each with different information requirements. To transition towards a more sustainable agriculture, inclusive educational programmes are important (Maini et al. 2021) and the knowledge intensive nature of addressing the challenges of food security, climate change and biodiversity means that education will play a critical role (Carlisle et al. 2019). Furthermore, the acceptance of the message is influenced by the relationship between the sender and receiver with acceptance increased where a favourable relationship exists (see S1 Fig. S1). As networks play a role in informing shared norms, values and understanding they can inform in part the attitudes towards messages received at an individual level. Whether messages are translated into actions is embedded in a complexity of factors encompassing aspects such as identity, knowledge, risk perceptions, belief, experience and aspirations (Leeuwis 2004).

Considering these dimensions and exploring networks, such as how information diffuses in the network could help to identify the opportunities for targeting. Networks with shared norms, values and understanding can facilitate cooperation and can generate bonding social capital between individuals and bridging capital, between groups or wider networks (de Krom 2017). In this sense, the role of social capital in social networks that farmers are associated with may be important for spreading information about practices and providing role models or generating norms of participation. Trust is an implicit feature of social capital (Coleman 1990; Putnam 1993) with the magnitude of network potential governed in part by the levels of trust between actors. Acknowledging the need to adjust approaches to knowledge exchange, learning and innovation in agriculture, already the EU EIP-AGRI interactive innovation model fosters a network-based approach to information exchange for competitive and sustainable farming and forestry across all actors in the agricultural knowledge innovation system (EC 2020a). Competitive and sustainable farming refers to a primary sector that can secure global food availability, provide a diversity of products and production, with greater farm profitability with supply efficiency and natural resource management reflective of environmental sustainability (EC 2012). In this regard, better understanding of the network of actors in the AKIS can help to identify actors to target with respect to specific agri-environmental objectives through incentives or other mechanisms.

### Aims and Objectives

In this research, we hypothesise that governance messages for soil functions converge at farm scale, which can result in overload of messages, making decisions related to sustainability challenging. The aim of this study was to increase understanding of messaging for soil functions in the network and the extent to which messages are accepted at farm scale. The major objective, using SNA and survey analysis data, was to expose opportunities to harness the potential of the network based upon network characteristics and farmer acceptance. The following research questions were addressed:

- What is the composition of current networks for soil functions in terms of context, diversity and scaling?
- What coverage of different soil functions is found in existing networks and to what extent are messages related to soil functions bundled, i.e. multi-functional?
- To what extent are messages related to soil functions coherent?
- To what extent are messages accepted by farmers?
- How can messaging for soil functions support sustainable land management?

## Materials and Methods

### Research Design

A mixed-method comparative case study design was used in this research. This included social network analysis (SNA) techniques. A social network is the structure of the interacting elements based upon a set of interconnected actors (nodes) and the connections (edges/arcs/ties/links) between them. Social network participatory mapping techniques in tandem with SNA were used to investigate the social structure of these interactions. In addition to social network data, participants each completed an individual survey to ascertain the degree of coherence and the acceptance or non-acceptance of messages. These data were compared with network data to reveal opportunities for targeting within the wider networks of the individual case studies.

### Selection and Overview of Case Studies

Three local case studies in three EU Member States were included in this research and were participants in an EU project exploring soil functions and the FLM framework. Lower Saxony in Germany; the southwest of Ireland and the Western Peat Meadows in the Netherlands. Case studies were selected based on several similarities and differences in relation to the agri-environmental governance systems. As EU Member States, these countries have shared priorities in relation to achieving EU agri-environmental targets but exhibit different levels of implementation and AKIS integration (Knierim and Prager 2015). The context-specific nature of networks means that the social networks are reflective of these local networks but not necessarily representative of the national pictures.

Farmland in the Dümmer-Lake region in Lower Saxony, Germany is used mostly for tillage (86%) with some grassland (13%) with 1% special cultivars. Many farms are mixed livestock and arable with one-third dairy or beef enterprises. Almost one-quarter do not have livestock and another quarter are pig or poultry enterprises. The Dümmer Lake is sensitive to phosphorus (P) eutrophication, so water quality is an important consideration in this area. The case study region in the southwest of Ireland is predominantly an intensive dairy catchment representative of the most intensively farmed dairying areas in Ireland. Most land is in grass and the area is characterised by herds producing milk under an intensive grass-based system. Nitrogen (N) loss via well-drained soils is the main threat to water quality in this area. The Western Peat Meadow Area is a typical Dutch landscape occupying large areas of the province of Utrecht, South Holland and the province of North Holland (Henkens 2013). Although a long history of dairy grazing farming exists, agricultural drainage and subsidence due to oxidation of the soil layer is a key concern in this landscape (Henkens 2013).

### Methods of Data Collection

#### Social network participatory mapping

Actor network data were collected between April and November 2017–2019 (Germany, *n* = 17; Ireland, *n* = 41 and Netherlands, *n* = 20). Social network maps representing the FLM governance network were constructed in participatory workshops. To understand the structural and functional aspects of how different actors conceptualise the governance network for soil functions, actors constructed network maps of the flow of messages related to soil functions. The main elements captured were nodes, which represent actors or organisations in the network with capacity (agency) to influence soil functions by sharing messages with other organisations and ties representing the links between nodes and messages for the five soil functions. During the mapping exercise, actors assigned probabilistic centrality tie weights calculated as the frequency (f) of a message multiplied by the likelihood to act (a) using the weight loadings shown in Table 1.

**Table 1:** Weight loadings for actor ties associated with frequency and action

Weight loadings (zero-order reciprocal approximation)			
Frequency (f)		Action (a)	
Daily	1	High potential—will transmit/act	1
Weekly	0.5	Med-High potential—should transmit / act	0.5
Monthly	0.25	Medium—may transmit/act	0.25
Annually	0.125	Low potential—unlikely to transmit/act	0.125
Less than annually	0.0625	Very low potential	0.0625

To complete the mapping exercise, participants were given an A0 sheet and sticky notes to populate the network. Following Burt (1983) and Reagans et al. (2003), respondents were asked a series of questions/prompts to generate nodes. To overcome issues of recall, a roster of ‘typical’ contacts was also provided (Reagans et al. 2003). The five soil functions were used as a formal criterion to boundary the network with analytical significance to the current research after Laumann et al. (1989). Prompt questions included:

Please compose a list of names of all the ‘organisations’ with which you share information in relation to your organisation (as information receiver or information supplier) for the soil function XXX?

Please consider all sources of information and knowledge – these can include your informal network—such as other farmers, family, social media, industry representatives, advisory etc.

Please also consider your formal contacts – such as the department of agriculture.

Prompts for individual soil functions were tailored to account for differences between actors in the network - so whether you are a farmer, or another actor. For example, in relation to water purification function, N, P and K application rates might be more pertinent for farmers, versus a prompt in relation to the Nitrates Directive when talking with policy-makers. All participants, along with those identified by respondents as message senders were included as actors and represent network nodes. Saturation was determined when all key actors listed prior to the sampling process based on literature and discussions with key informants appeared in the network. Previously, the Pro-AKIS project had visualised the main actors in the AKIS that link people and organisations at MS level, providing an overview framework (Pro-AKIS 2013). While the earlier work provided insight into the AKIS in general, in this research we distil the networks out of our analysis of who is sending messages to whom in three case studies in relation to five soil functions.

In addition to relational data, respondents were also asked to fill in node attribute data to describe the type of organisation (farm, business, government, NGO, R&D, other, unknown) and the scale of the node (local, county, regional, national, international or unknown). To eliminate bias associated with power relations, the egocentric data for key actors were collected and combined to produce egocentric networks by actor type, e.g. farmer network. These were subsequently combined into a single data matrix to visualise the whole sociocentric network. Where repeat ties occurred between nodes, these were merged in Gephi © 9.2 software and reflected by the average tie weight. This meant that actor types were provided equal weighting when ties were subsequently generalised to reflect the whole network to avoid an actor bias. Network statistics were calculated for the whole network based upon the combined SNA maps within case studies. Two facilitators completed the data collection following a guide for data collection to ensure consistency. Two preparatory meetings prior to data collection took place in the Netherlands in 2018 to ensure facilitators were prepared to engage in a consistent, accepted, productive and neutral way.

#### Survey data

Respondents answered questions to characterise the messages they receive on the basis of consistency and coherence. They were also asked about how to improve coherence in the messaging they receive. Farmer respondents indicated which actions they have implemented on farm, structured along mandatory, market or voluntary measures to provide insight into sustainability actions at farm scale and the key message source for measures implemented. This represents the operationalisation of message (non-)acceptance. The source of messaging for measure implemented was recorded also to understand who the key brokers were and to allow for a comparison between them and those identified as the most central actors in the whole network.

### Methods of Data Analysis

#### Social network analysis

Gephi © 9.2 software was used to visualise and analyse the networks using exploratory SNA techniques. Exploratory network analysis methods are based upon graph theory whereby maps are transformed into adjacency matrices with nodes listed on the horizontal and vertical axes. A weight is coded in the matrix where a tie is observed between two nodes (Scott 2017); in this instance weighted ties were used. The metrics used to analyse network position and for network structure are shown in Table 2.

**Table 2:** Numerical expressions used to assess and compare the social networks of each case study region and structural properties

Node properties			
Node position	Numerical expression	Definition	Node characteristic(s)
Out degree (OD)	$ODi = {{\Sigma }}\left| {a_{ij}} \right|$ k=1	The cumulative strength of connections with which a node influences others	Driver
Weighted out degree (WOD)	$ODw = {\sum} {\left| {a_{ij}} \right|} \ast {\sum} {Wa_{ij}}$ k=1	The out degree of a node considered by the total weight of its outward edges	Influencer
In degree (ID)	$IDi = {\sum} {\left| {a_{ij}} \right|}$ k=1	The cumulative strength by which a node is influenced by others	Receiver
Weighted in degree (WID)	$IDiw = {\sum} {\left| {a_{ij}} \right|} \ast {\sum} {Wa_{ji}}$ k=1	The in degree of a node determined by the total weight of its incoming edges	Affected
Centrality Degree (D)	$DoC_i = OD_i + ID_i$	The cumulative strength of connections of a node (in and out ties)	Central
Weighted degree of centrality	$DoC_{i\,w} = OD_{iw} + ID_{iw}$	The degree of centrality of a node determined by the total weight of all its edges	Dominant centrality
Betweenness Centrality	$C_B\left( {n_i} \right) = {\sum} {g_{jk}\left( {n_i} \right)/g_{jk}}$ j < k	The fraction of shortest paths that go through a node divided by the total number of shortest paths between nodes	Broker/Bridge
Closeness Centrality	$CC\left( i \right) = \frac{{N - 1}}{{{\sum} {d\left( {i,j} \right)} }}$ *j*	Average length of the shortest path between node and all other nodes.	Diffusion
Network Structure			
Number of nodes	*N*	The number of components in the map	
Number of edges	*E*	The total number of linkages between components	
Diameter	$D = \mathop {{\max }}\limits_{st} \left\{ {a\left( {s,t} \right)} \right\}$	The shortest path length in the network (the shortest distance between the most distant nodes in the network).	Indicates how long it would take (or how many intermediary nodes it will take) for messages to circulate between the two most distant nodes.
Density	*Dn* = *E/N(n−1)*	Indicates how densely nodes are connected	

In Table 2, *a* represents each edge, *i* is the transmitter node of edge *a, j* is the receiving node of edge *a* and *W* is the weight of edge a (example source calculation references see e.g. Özesmi and Özesmi 2004; Micha et al. 2020). For betweenness centrality, *gjk* represents the number of geodesics or shortest paths connecting *jk*, and *gnk (ni*) = the number that node *i* is on (based on Derrible and Holme 2012). For closeness centrality, where I ≠ j and dij is the length of the shortest path between nodes i and j in the network, N is the number of nodes following Sabidussi (1966). Diameter *a*(*s*,*t*) indicates the number of edges in the shortest path from a node *s* to a node *t*. Density represents the number of edges (*E*) divided by actors times actors minus 1.

In addition to network properties and structure, ties were analysed in terms of the soil functions that they applied to so that the coverage of different soil functions in the network could be established along with the bundling capacity of network actors.

Survey data were analysed using Microsoft excel 2016 to determine acceptance of messages based upon the translation of messages received into action. Survey data were compared with network analysis results in terms of similarities or divergences. This comparison exposed opportunities for future targeting of actors in the wider network described in the SNA.

## Results and discussion

### Social Network Analysis

#### Network compositions and characteristics—context, diversity and scale

Data collected through the participatory mapping exercises are used to describe the composition and characteristics of the network and are presented in Tables 3, 4 and 5. Table 3 shows the global structure of each network such as size, diameter and density. Table 4 shows the types of actors in the networks while Table 5 shows the scale of actors.

**Table 3:** Global network context of the case studies

Network	Germany	Ireland	Netherlands
Nodes (actors)	52	110	73
Edges (ties)	130	259	213
Diameter	4	5	4
Path length (average)	2.37	3.278	2.605
Clustering Coefficient (average)	0.175	0.07	0.142
Graph density	0.049	0.02	0.041

**Table 4:** Soil function sustainability network diversity—actor type

Case study	Type												
	Farm Org	Business	Governmental	Semi-state	NGO	Research	Advisory	Info	Lobby	Multi-actor	Other	Total nodes	Total ties
Germany	5 (10%)	17 (33%)	10 (19%)	2 (4%)	5 (10%)	1 (2%)	7 (13%)	2 (4%)	2 (4%)	1 (2%)	–	52	130
Ireland	3 (3%)	30 (27%)	45 (41%)	2 (2%)	3 (3%)	6 (5%)	7 (6%)	2 (2%)	1 (1%)	3 (3%)	8 (7%)	110	259
Netherlands	5 (7%)	14 (19%)	12 (16%)	1 (1%)	7 (10%)	11 (15%)	2 (3%)	2 (3%)	5 (7%)	4 (5%)	10 (14%)	73	218

**Table 5:** Number and percentage of organisations per scale in the network—scaling potential

	Local	County	Regional	National	International	Multi-scale
Germany	11 (21%)	7 (13%)	13 (25%)	9 (17%)	4 (8%)	8 (15%)
Ireland	13 (12%)	1 (1%)	14 (13%)	67 (60%)	15 (14%)	–
Netherlands	14 (19%)	1 (1%)	14 (19%)	38 (53%)	3 (4%)	2 (3%)

In relation to messages for soil functions, the largest sustainability network was identified for Ireland (110 nodes and 259 edges) compared to the German case study, which had the fewest nodes and ties (52 nodes and 130 edges) (Table 3). Having a larger network can foster innovation but this also relies on the diversity of these actors (Hermans et al. 2017). Graph diameter is a measure of the shortest distance between the two furthest actors. Graph diameters of four, five and four were found for Germany, Ireland and the Netherlands respectively. In practice, this means that for messages related to soil functions to be transmitted between the two most distant actors the messages would need to transit between three, or four in the case of Ireland, other actors. This is an indication of small but highly connected networks (Iijima and Kamada 2017). Average path lengths of ~40% less than graph diameter indicates a relatively high network efficiency. While most nodes are not directly connected, only a few intermediary steps exist between any two nodes. A relatively low diameter, coupled with a power law distribution (meaning the presence of a few dominant hubs with very high degree representing a high number of messages) (S1, Fig. S2) are characteristics consistent with the presence of ‘small worlds’ (Travers and Milgram 1969; Watts and Strogatz 1998) and were found in all case studies. The emergence of some actors with high clustering coefficients further suggests cliques whereby these particular nodes have dense networks with friends who tend to be friends with one another. In Germany, examples include a hunting organisation or the dung board. In Ireland, the food marketing board and soil analysis contractors have these characteristics while in the Netherlands it includes the young farmers lobby group. These may be effective actors for relaying information while keeping the number of links to connect the network to a minimum. However, this must be considered within the global network context, whereby low average global clustering coefficients indicate a relatively low cohesion in the networks overall with only about 15% of possible triads (triangles between three nodes) completed (Table 3). Furthermore, graph densities < 1 were found in each of the case studies (all < 1 where 1 indicates all nodes are connected to each other), indicating network vulnerability and that potential for increased connectivity in the networks exists.

Table 4 shows how diverse the networks are, based upon the types of actors represented. Diversity in networks is an important indication of the potential for innovation as different actors bring different understandings into the biophysical, technological and institutional dimensions of the problem with capacity to innovate benefitting from interactions between a diversity of actors (Hermans et al. 2017).

The networks analysed here reflected a broad mix of different actor types with private business and public governmental actors the most prevalent types of actors across all networks. Thereafter, actors described as research or advisory were the most identified actor types in these networks (Table 4). In this regard, each of the networks reflects a diversity of actors with no noticeable absences of a particular actor type observed.

The spatial scale at which actors operate provides insight into the scaling potential of networks. Table 5 shows the proportionality of organisations per network scale. Actors at different scales typically have access to different resources such as knowledge or power. Networks with connected actors operational at different administrative scales are relevant so that information or other resources can flow between different levels (up-scaling) (Hermans et al. 2017).

The German network unveiled a more even distribution of actors at different scales (Table 5). Regional scale actors accounted for one-quarter of nodes, followed by local scale actors (21%). In relation to the political geography, the networks highlight two main differences. In Germany, a much higher proportion of the network is assigned to ‘county’ scale while the ‘national’ scale is far higher in the other case studies. The much larger geographic spatial scale is likely a driver of the key differences between Germany and the other case studies. In part, this may be due to the respective EU institutional frameworks of the case studies, administered at regional scale in Germany due to federal governance potentially more efficient, compared to the national scale in Ireland and the Netherlands where much higher proportions of national scale actors are reported. Even though all countries are within EU implying a similarity in agri-environmental governance, the island nature of Ireland may be important with the high proportion of national scale actors linked with a greater need for connectivity at wider geographical scales. Beyond actors at different scales, the presence of multi-scale actors represents added opportunity to connect administrative scales. Actors described as ‘multi-scale’, such as the dairy industry or a farmer association were recorded in Germany (15%) but were not described in the Irish network with a low proportion identified in the Dutch case study (2.7%).

While there is no one optimal network configuration, in relation to scaling and innovation potential as per Hermans et al. (2017) the German network theoretically reflected greater scaling potential based on the network configuration whilst the larger network found in Ireland may have greater scope for innovation due to the larger network of actors reported.

#### Who’s Who? Actor prominence

Different measures of centrality are useful for understanding the role of different actors in the network. Within networks, different actors occupy different positions with respect to the level of prominence that they hold associated with the volume of messages that they both receive and emit (degree) or that they receive (in-degree) or emit (out degree). A high degree centrality means that an actor receives and emits a high number of message making then central to the network. Table 6 provides an overview of the position of the top ten actors in the networks based upon centrality measures allowing the most important actors in the network based upon the number of connections to be identified. Those with a high weighted in degree (WID) receive a lot of messages whereas those with a high weighted out-degree (WOD) emit a lot of messages and are also referred to as influencers. Actors with high out degree can exchange with many others and can make people aware of their views, which increases the capacity of these actors to be influencers in the network.

**Table 6:** Whole network centrality results for case studies

Germany									
Actor	Degree	Actor	WID	Scale	Type	Actor	WOD	Scale	Type
Germany									
NGO (SustAg)	51	NGO (SustAg)	7.61	R	NGO	NGO (SustAg)	7.66	R	NGO
Farmer	47	Farmer	7.21	L	F	Farmer	7.59	L	F
Advisory (CALS)	35	Advisory (CALS)	3.78	MS	A	Advisory (CALS)	2.35	MS	A
Agricultural Contractor	6	Advisory (LWK)	1	MS	A	Natural Resource Management	1.1	R	G
County Council	6	Natural Resource Management	1	R	G	Advisory (LWK)	1	MS	A
Advisory (BRS)	6	Research (University)	1	N	R	Research (University)	1	N	R
Environment Association	6	Family	1	L	F	Family	1	L	F
Advisory (LWK)	6	Other Farmers	1	L	F	Other Farmers	1	L	F
Environment (NLWRN)	5	Inputs (seed)	1	L	B	Inputs (seed)	1	L	B
Water Supply (WSV)	4	Agriculture Ministry	0.75	MS	G	Weather	1	N	I
Ireland									
Farmer	52	Farmer	5.70	L	F	Fertiliser Industry	6.13	R	B
Fertiliser Industry	36	Fertiliser Industry	5.07	R	B	Farmer	4.71	L	F
EPA (Catchments)	31	Environment Agency (EA) (Licensing)	4.69	N	G	EPA (Licensing)	4.44	N	G
EPA (Licensing)	30	Advisor (Teagasc Specialist)	3.98	N	A	EPA (SEA)	3.39	N	G
DAFM Climate	30	EPA (Catchments)	3.18	N	G	EPA (Catchments)	2.78	N	G
EPA (SEA)	25	DAFM (Climate)	2.52	N	G	DCCAE – Climate and Environment	2.46	N	G
Dairy co-operative	22	Dairy co-operative	2.23	L	B	Research (Teagasc)	2.39	N	R
Research (Teagasc)	22	Research (Teagasc)	1.57	N	R	Advisor	2.37	N	A
EPA (SOE)	20	DAFM	1.48	N	G	Dairy Co-op	1.81	L	B
DAFM	17	Media (online)	1.21	N	I	DAFM	1.72	N	G
Netherlands									
Farmer	72	Farmer	9.19	L	F	Farmer	7.42	L	F
Nature Board	46	Nature Board	6.27	N	MA	Nature Board	6.36	N	MA
Research	43	Research	5.96	N	R	Research	4.46	N	R
Gov (Reg)	35	Agriculture & Nature Collective	3.02	P	MA	Agriculture and Nature Collective	3.57	P	MA
NGO (SustAg)	22	Regional Government	2.39	P	G	Regional Government	2.31	P	G
Agriculture & Nature Collective	16	NGO (Sustainable Agriculture)	2.23	N	NGO	NGO (Sustainable Agriculture)	2.23	N	NGO
Water Board (Regional)	12	Water Board (Regional)	1.57	P	G	Nature Lobby	1.55	N	L
Nature Lobby	10	Local Dwellers	1.50	L	O	Local Dwellers	1.5	L	O
Advisor (P)	7	Nature Lobby	1.32	N	L	Media	1.33	N	I
University	7	Services – AI	1	L	B	Farm workers	1	L	F

The farmer represented the most central actor in Ireland and the Netherlands, with the highest degree centrality based upon the number of messages (received and emitted) (Table 6). This confirms that most messages converge at farm scale. As more farmers completed the network data collection exercises, this plausibly increases scope to identify more actors and in turn can increase centrality scores even if ties were averaged for all farmers in a given network. However, this result is still consistent with previous findings in relation to farmer stakeholders who are considered to have a high degree of centrality for agricultural biodiversity governance (Hauck et al. 2016). In Germany, an NGO on sustainable agriculture had the highest overall degree (received and emitted). The farmer was the second most central actor in the German network, still indicating the prominence of farmers in the network for agri-environmental messages related to soil functions with a high message load converging at farm scale. In the Netherlands, the farmer represents the actor who both receives the most messages but also has the highest Weighted Out Degree (WOD) indicative of high capacity to influence. In Ireland, the fertiliser industry emerge as having high potential to influence and in Germany the NGO working in sustainable agriculture held this position. These actors are important given their potential to influence the network, however, it must be borne in mind that the extent to which this potential is utilised will rely on the extent to which messages might be congruent with the central objectives of these actors.

**Fig. 1:**
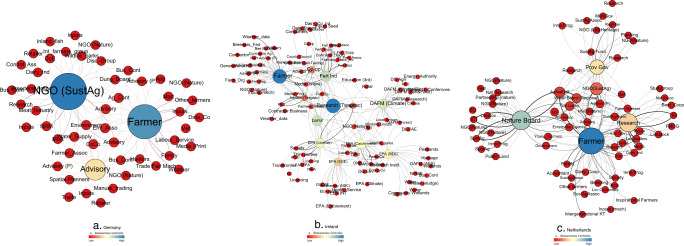
Betweenness centrality indicative of key bridging actors in the network. The most important bridging actors are shown in blue

In addition to degree centrality, betweenness centrality was analysed (Fig. 1). Betweenness is important to identify the bridging actors in the network as they can influence flow around the network. Betweenness centrality is based upon the number of shortest paths that pass through an actor (Freeman 1977) and is a critical measure to assess which actors could act as bridging agents in the network. In the absence of these actors, different actors in the network would be disconnected so these actors are important brokers to reach certain actors.

Across the networks, farmers are a top bridging actor (Fig. 1). Consistent with previous findings, sustainability organisations/collectives are important in the Netherlands and Germany (Prager 2015) whereas governmental bodies and industry partners represent the key brokers in the Irish network. While having the potential to act as a bridge or knowledge broker, these actors may also be gatekeepers of information. The power of this role may give rise to a service charge. Maury et al. (2013) emphasise attention for actors who bridge gaps between environment, agriculture and territory that through their presence, may have potential to mobilise resources particularly in relation to coordination. This may be particularly important going forward as there is heightened demand to validate the sustainability credentials of agricultural production. Meeting targets outlined under emerging policies, for example a 20% reduction in fertiliser (EC 2020), is likely to require many actors across the whole chain, who may evolve as having a role in coordination, for example, industry partners. In some instances, additional bureaucracy could be potentially off-set where such coordination could accrue benefit via marketed goods that could be linked to a reduced footprint in relation to environmental outcomes.

**Fig. 2:**
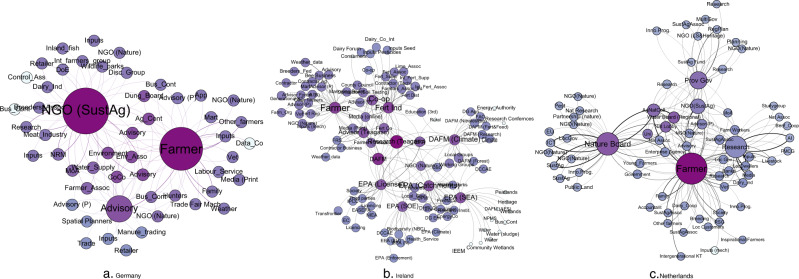
Closeness centrality whereby actors with highest closeness centrality (darkest colour) have potential for message diffusion in the network

Closeness of a node represents the shortest distance between a node and all other nodes. Closeness highlights those actors that can readily influence the network quickly and are useful for information diffusion based upon their proximity to other actors, as shown in Fig. 2. Closeness centrality is important as these actors can perform a role in information diffusion in the network due to their capacity to reach actors more readily than actors that are further away.

In general, those actors that had high betweenness were also characterised by high closeness centrality (Fig. 2). In Germany, the NGO, the farmer and the advisor had high closeness centrality (Fig. 2a). This was similarly the case in the Netherlands (Fig. 2c) where farmers and the nature and sustainable agriculture organisations emerged as close. The closest actors in the Irish case study were mainly government or national bodies such as the Ministry for Agriculture or the public agricultural research authority (Fig. 2b). Where this value is zero is indicative of a node that is disconnected and infinitely far from some nodes. Some of these actors were found in Germany (*n* = 3 e.g. data company) and the Netherlands (*n* = 1 e.g. mechanical inputs supplier), however, there were several found in Ireland (*n* = 17) and examples included the energy authority or international conference fora.

### Targeting Messages

#### The nature of bundling—who and at what scale

**Fig. 3:**
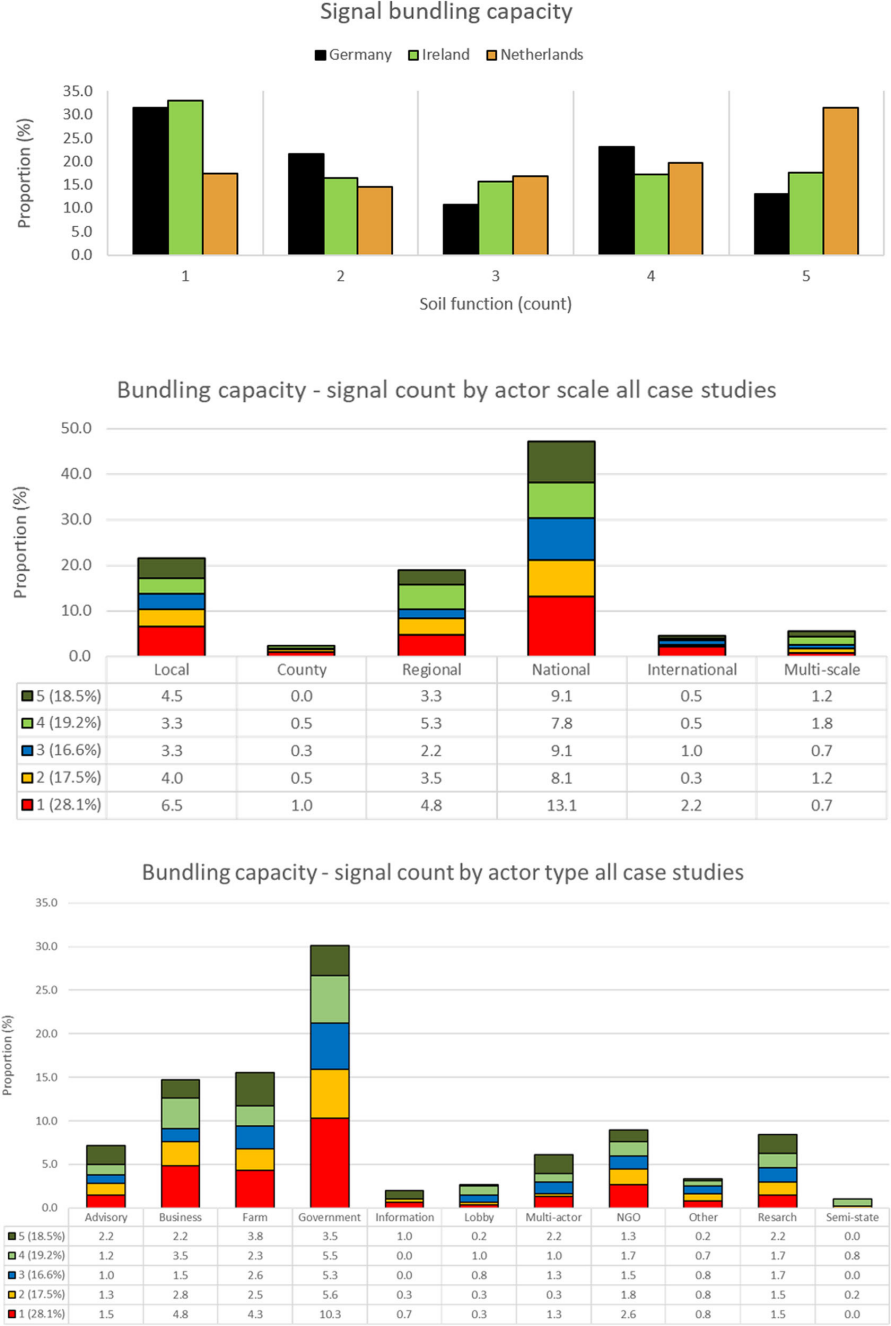
Message bundling graph showing the distribution of ties and the respective number of soil functions captured by country (top), by scale (middle) and actor type (bottom) across all case studies

At a practical level, farmers occupy a key role in the implementation of practices and measures for sustainable land management. As the suite of competing demands on land is growing, other actors who communicate messages related to soil functions could be targeted to bundle and align messages to help reduce the complexity at farm scale. This requires in the first instance understanding of the extent to which different network actors are already bundling messages. Figure 3 shows the current extent of bundling or multi-functionality within the networks by country, by scale and by actor type.

A greater proportion of actors in Dutch network transmitted messages that encompassed all soil functions (Fig. 3, top). This indicates that multi-functionality and more holistic approaches to sustainable land management are more prevalent within the Dutch social network. In contrast, the German and Irish networks had a similarly high proportion of messages wherein messages related to just one dimension of soil functionality. These network compositions include higher proportions of business and governmental actors and messages exchanged thus reflect the more specialist nature of business exchanges or policies that promote certain business exchanges or a lack of horizontal integration with respect to policies related to soil functions. However, these networks did have actors whose messages were more multi-functional. In Ireland, the public agricultural research authority showed to be a key actor for messages that related to all soil functions reaching multiple other network actors such as industry partners (dairy co-operative, fertiliser industry), farmers and advisory services (public, specialist and private). In Germany, farmer ties with other farmers, family, advisory, along with ties between the sustainable agriculture NGO and advisory captured the full suite of soil functions.

Collectively, with respect to scale, a mixed pattern emerges whereby actors at all scales reflect varying degrees of bundling capacity (Fig. 3, middle). While the largest proportion of messages emitted by national and local scale actors are only in relation to one function (28.1%), the next largest proportion of messages at the same scales relate to all five soil functions. Despite this mixed outcome, while some national scale actors have a key role for bundling, as they are highlighted as key message emitters in general, it means that overall they can have a disproportionately large role with respect to bundling of messages to reduce message complexity and overload at farm scale. As for the bundling capacity of particular actors, farmers emerge as the number one actor that transmits messages related to all soil functions (Fig. 3, bottom). However, as a proportion of the messages by actor type, advisory, information and research actors along with actors defined as multi-actor are indicated as having the highest bundling capacity with respect to the messages that they emit. As the demand for multi-functionality grows, actors with information across multiple domains may have growing importance as brokers in future.

#### Tie strength

While it is important that while a tie between actors might be multi-functional, the message strength (based upon weighted influence) of that tie provides insight into whether actors are more or less likely to use that information.

**Fig. 4:**
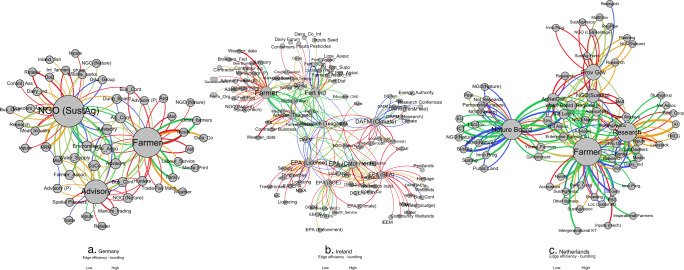
Tie strength indicated by width of tie between actors with widest ties representing the strongest ties. Bundling shown by tie colour from 1 soil function (in red) to 5 soil functions (in green)

In Germany, other farmers and family are the key actors with whom farmers exchange the strongest messages related to all soil functions (Fig. 4). This is consistent with homophily whereby actors preferentially identify with actors having the same traits and are more likely to form ties (Rogers 1983; Newman 2003). Strong ties between actors that are similar in a sociodemographic sense are characteristics associated with trust and bonding social capital (Szreter and Woolcock, 2004; Klerkx and Proctor 2013). Where homophily exists, the likelihood of more rewarding communication increases due to shared common meanings (Rogers 1983) but the limitation with respect to innovation potential that this might represent must be borne in mind as greater heterophily typically adds more capacity for innovation. In the Irish network, strong ties are reported for inter-agency actors in the environment sector, but also, with environmental consultants. Strong ties also occur between actors who are less similar, for example between an input provider and the farmer with the message specific to only one function (Fig. 4). This tie represents a formal collaboration with a brokerage function that is important to the farmer but only addresses a singular aspect of soil functioning. In Ireland, the use of weather apps for weather information was amongst the strongest message for farmers that influenced their decisions related to primary productivity. However, for all soil functions, the fertiliser industry relayed strong messages to farmers with other strong messages between public advisory and farm relief services, or farmers and public specialist advisors. In the Netherlands, strong ties occur between actors engaged in conservation and sustainable agriculture (e.g. nature board, sustainable agriculture NGO, agriculture nature collective). Farmers also have strong messages with the same collective while research actors are strongly connected with business actors (artificial Insemination), media actors and local dwellers.

### Targeting Soil Functions in Favour of Sustainable Land Management

#### Coherence requirement and needs

Our results confirm that a large number of messages converge at farm scale which can result in overload and difficulty for decision-making related to sustainability. To validate the latter, survey respondents were asked whether decision-making related to sustainability is difficult, whether messages come from multiple sources and to what extent messages are conflicting or coherent. This allowed for the establishment of a composite indicator to highlight to what extent a need for greater coherence within messaging exists. In general, across all case studies farmers indicated a need for greater coherence (Germany 66%, Ireland 56% and Netherlands 52%). While 20% of farmers neither agree nor disagree on a need for more coherent messaging, one-quarter indicated a low requirement for greater coherence in the Irish case study, which increased to 28 and 39% in Germany and the Netherlands respectively. The latter result coincides with the greater multi-functionality of messages observed in the Dutch network analysis even if a sizeable gap still exists. As to how to increase coherence, more face-to-face interaction and discussion groups emerged as important and while more advisory was indicated for the Irish case study, the existing level of advisory was considered sufficient in the other case studies. Online platforms and group apps were proposed as potential support tools in the Netherlands. The highest demand overall was for more discussion groups endorsing the importance of peer-to-peer interactions and the high value placed on connections associated with trust and bonding.

#### Action implementation and message acceptance

**Fig. 5:**
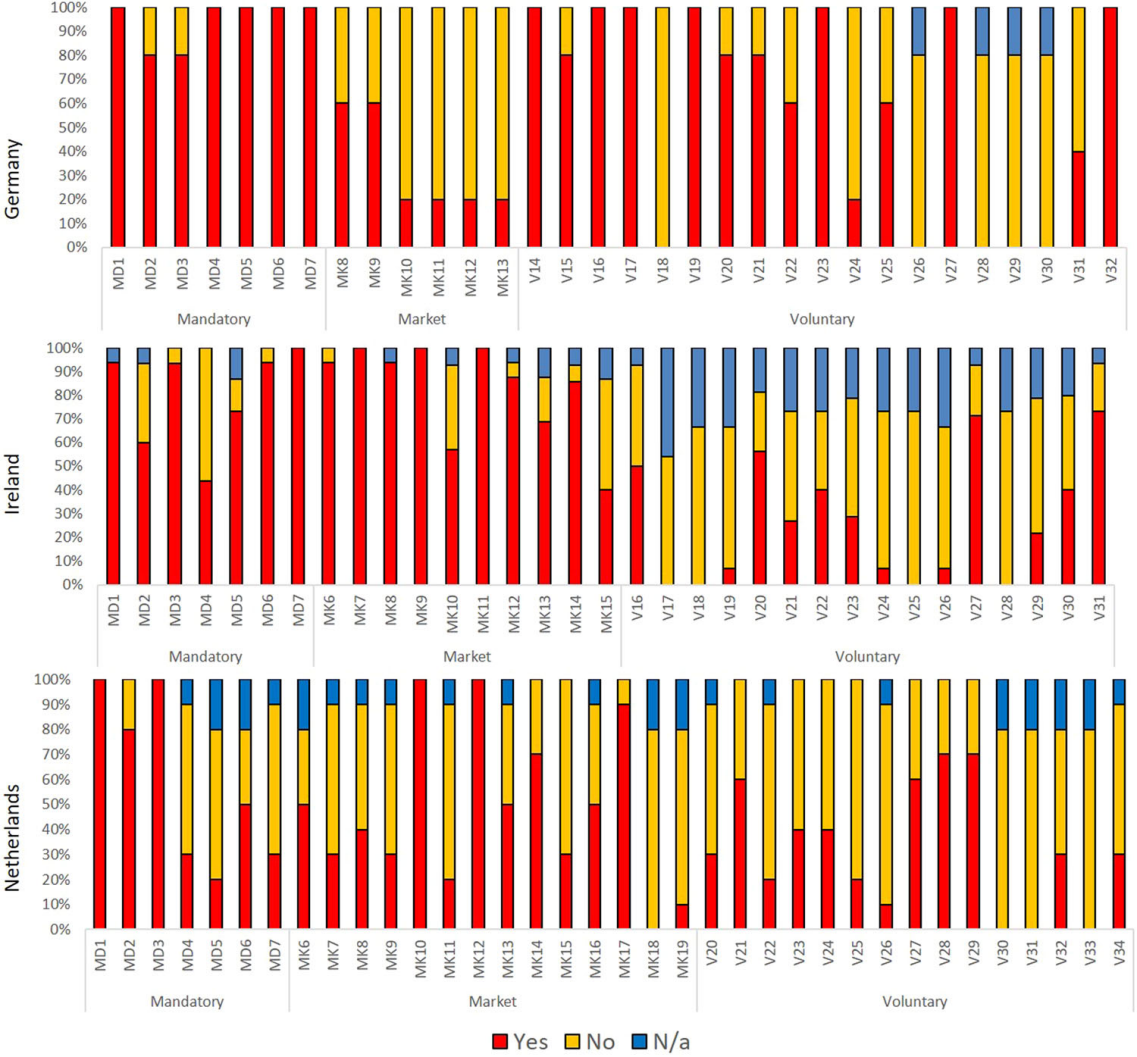
Proportional uptake of measures at farm scale structured along mandatory (MD), market (MK) or voluntary (V) instruments for case studies as indicated by farmers. For a full list of measures, see Supplementary Information S1, T1

Figure 5 shows the proportional uptake of measures grouped according to their nature, whether mandatory, market or voluntary to provide insights into actions taken at farm scale. This insight into sustainability actions at farm scale helps to better understand which messages farmers are more responsive to. It also operationalises the non-acceptance of messages in the network, which may be particularly relevant for emerging policies that may or may not resonate with farmers, falling inside or outside their latitudes of acceptance.

The measures or actions implemented by farmers in the German and Irish case studies are orientated in favour of compliance requirements (Fig. 5). Thereafter, in Germany a prioritisation of soil protection and management in terms of the uptake of voluntary actions by farmers is reflected in the results (V14 soil testing, V16 and V19 erosion related measures, and V17 fertiliser training and V23 use of low emission slurry spreading) and is consistent with regional objectives to support water quality in Lower Saxony. In Ireland, selected voluntary measures included the use of low emission slurry spreading (V27), fencing off watercourses (V20) and integrated pest management (V31). The farmers in the Irish case study were a group of intensive dairy producers and measures selected more directly related to agricultural practices and a need to support the sustainability of their enterprise. Land management in favour of farmland birds is a high priority for Dutch agriculture (EC 2020b) and was reflected in market and voluntary measures (MK10 Conservation of meadow birds, V27 Management of breeding primary meadow birds and V28 Management for other birds) that had a high uptake at farm scale. The implementation of buffer strips (MD4) had a low uptake but reasons for this have been described previously, including the high productivity of agricultural field margins, the potential increased need to export manure and loss of eligible land under CAP area based payments (Dworak et al. 2009). With respect to the voluntary measures, implementation reflected the particular challenges specific to the case studies. Awareness of the context-specific environmental challenges in their areas may have prompted farmers to act, as greater environmental awareness has previously been found to support the uptake of agri-environmental schemes in the UK (Beedell and Rehman 2000; Wynn et al. 2001; Baumgart-Getz et al. 2012).

Overall, there is a high proportion of measures that were not implemented or were considered as not applicable. Reasonably, capacity and resources to implement all measures everywhere is not plausible. In general, farmers that are more commercially orientated may require larger economic incentives to switch from more intensive production systems that benefit from higher economies of scale from food production versus the return on environmental services (Gailhard and Bojnec 2015). For example, the Irish farmers who participated in the survey are more intensive producers and engendering a shift towards voluntary schemes would require sufficiently competitive schemes relative to market or compliance requirements for these farmers. Although financial incentives are important in relation to uptake of schemes and practices (Posthumus and Morris 2010), alone they overlook intrinsic motivations which may diverge from incentive objectives resulting in low adoption of proposed measures, irrespective of financial rewards (e.g. Duesberg et al. 2014; Greiner and Gregg 2011). It is also important that existing lock-ins be considered. Farmers in the Dutch case study indicated diverse system lock-ins that impede sustainable management practices that were not only motivated by profit maximisation objectives, even if they are willing to implement them (de Vries et al. 2019).

Although awareness has been associated with the enhanced uptake of measures, it may not necessarily increase the acceptance of messages in favour of action. For example, the Dutch and Irish case studies reported a lack of recognition of existing sustainable land management practices. In both case studies, actors indicated that society attributes a disproportionate responsibility on agriculture for environmental challenges compared to other actors and sectors, indicating an awareness of the need for sustainable land management. At the same time, this had resulted in a polarisation of ‘them’ and ‘us’ occurring, which could reduce acceptance of messages (de Vries et al. 2019). Expanding market-based initiatives (MBI) to incorporate a wider range of targeted public goods for which consumers are willing to pay may be another pathway that could share responsibility more widely however, with respect to the case study network for soil functions, there was limited reference to consumers and their role in these networks. Market instruments act to correct market failure associated with negative externalities and are designed to address some form of pricing signal (Cocklin et al. 2007) and can include negative (e.g. tax) or positive (e.g. subside) incentives (Pannell 2008). The high cost of regulations makes MBIs an attractive way to reach conservation objectives more cheaply as they use market forces to pass on incentives while also have potential to generate revenue that can be funnelled towards conservation management, acting as a complementary approach rather than an alternative (Bräuer et al. 2006). This could offer a compromise between intensive production and environmental services making the latter a more attractive alternative.

In general, messages that fall outside the latitude of acceptance that are too far from farmers’ internal logic or aspirations are more likely to be rejected or filtered with a multitude of socio-economic and cultural factors impacting the acceptance of a message (Leeuwis 2004). These findings highlight important considerations, particularly for those measures that were rejected or considered ‘not applicable’, for example organic farming which is becoming a heightened priority in the emerging policy landscape under the European Green Deal (EC 2020). Increasing uptake would require combined policy mixes that in addition to incentives or market signals would require education and information tools and potential re-framing to increase the acceptability requiring participation of actors all across the agri-food chain. A deeper assessment into the acceptance of messages in the Dutch case study found that the relationship between the farmers and their network was important for acceptance of messages and recommended placing effort towards establishing relationships between farmers and their network (de Vries et al. 2019). Multi-actor approaches, such as operational groups are an opportunity for AKIS actors to work together to find solutions to overcome gaps and bottlenecks that draw on tacit knowledge and participation that in principle could also serve to increase acceptability.

### A Comparative Analysis for Targeting Opportunities

**Fig. 6:**
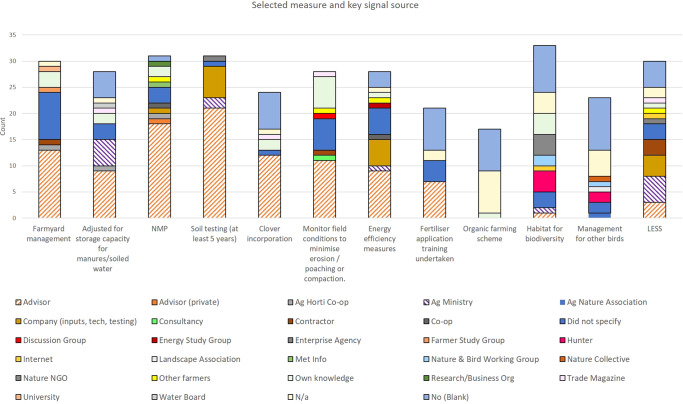
Selective universal measures and the main message source as identified by farmers for all case studies

A comparison between survey results and the SNA allowed us to see to what extent influential network actors in the global networks were consistent with the key actors that farmers engage with for the implementation of measures on farm (shown collectively for some common measures Fig. 6). From this, we can identify opportunities or challenges that exist within the current network for targeting or bundling of messages.

Despite the assigned influence of certain actors in the SNA, their influence did not always translate into action at farm scale. In Germany, advisory emerged as the key signal emitter with respect to actions implemented on farm (50%), followed by messages from other farmers (21%) the latter consistent with the SNA. The SNA indicated weak messages from advisory, but in practice, these emerge as the most important broker at farm scale with farmers most likely to act on a signal received from an advisor over other actors. So, while an NGO on sustainable agriculture had high prominence in the SNA their signal ties with farmers were not strong and only 3% of signals associated with implementation were from a nature NGO. These results are consistent with other research whereby the message origin may be more decisive than the information itself (e.g. Ulrich-Schad et al. 2017). This is also found in the Irish network, whereby the fertiliser industry emerged as having high bundling capacity with potential to influence, but were not highlighted as a key message source for the implementation of measures at farm scale with survey results highlighted that advisory occupyied that role. This result is consistent with very high levels of trust indicated by farmers in their advisor, even at the early stages of the relationship due to trust in the organisation (Teagasc) (Gorman et al. 2019). Altogether, the results point to message overload with many messages in the network not translating into action. They also highlight the complexity of decision-making at farm scale. In the case of the fertiliser industry in Ireland or the German NGO who have high bundling capacity, messages were not prioritised by farmers and may be filtered or lost or are simply superseded by messages from more established trusted brokers. The nature of the relationship between actors has an important role in the receptivity of messages exchanged. As a need for coherence was indicated, this can also explain why messages of some actors are filtered where an already large message load converges at farm scale. In many instances, key brokers already have a high bundling capacity but this is not always the case. From this, we can determine different potential pathways for targeting. On the one hand, actors whose ties have high bundling capacity or network influence could be targeted by strengthening such relationships and building trust through greater collaboration. Alternatively, the remit for actors whose ties have high weight that have shown to inform implementation but only refer to one aspect of soil functioning could be expanded. For example, weather apps are an important information source for farmers to support their decisions around primary productivity but could potentially integrate other information that is of relevance for farmers for sustainability. The use of a farm sustainability tool (FaST) for nutrients under new good agri-environmental condition (GAEC) requirements was proposed at EU level, and is consistent with the Dutch case study who indicated a demand for more online/app tools. Business partners often have these characteristics also as they are providing a paid service demanded by farmers usually with respect to a particular topic. Fundamentally, targeting existing relationships that show a high level of trust exhibited through current implementation practices may have more immediate potential than building new relationships.

## Limitations and Further Research

This work has looked at three key studies. As such, networks are context specific and therefore not generalisable. This has not been the purpose of this work but rather to see what insights can be garnered that have wider relevance that could support greater sustainability in the future. In this regard, we have tested our hypothesis in three different contexts that highlight both commonalities and differences and we have advanced our understanding of messaging, and opportunities to effect change towards sustainable land management. Future research should consider different farm typologies as different farming systems and intensities are likely to require differentiated targeting of messaging. Furthermore, the similarity of message content between entities as this was not explored here and may have importance to increase coherence and efficiency within the networks if better understood. As shown here and consistent with other research, awareness, acceptance and ultimate adoption have a high dependency on multiple factors such as farm and farmer characteristics, value and belief systems, and economics.

## Conclusions

SNA techniques revealed a diversity of actors in the AKIS with actors forming network links with different types of organisations and at multiple scales in each of the three case studies. The larger network in the Irish case study suggests potentially greater capacity for innovation while the regional configuration generated greater scaling potential in the German case study. Farmers showed to occupy a central role in the agri-environmental governance, as evidenced by the convergence of messages at farm scale. This in turn can increase the complexity for making decisions for sustainability with respect to the implementation of actions and measures on farm. A greater level of multi-functionality in the Dutch case study coincided with a reduced requirement for coherence within messages. This indicates that greater attention to increase multi-functionality across actors within the AKIS can help to support enhanced acceptance, which could in turn support greater sustainability. Awareness and acceptance of messages showed to be important. Particularly important, was the reliance of farmers on trusted brokers. In this regard, the amount of information became less relevant than how information was received. This was further endorsed with respect to the coherence requirements specified, with a preference for peer-to-peer interactions rooted in trust and bonding. The main lessons learnt are as follows:

- Farmers indeed receive an overload of information; this results in low acceptance of messages in the network.
- The signals that are acted upon and translated into farm management actions depends in the first instance on the source of the message, rather than their content per se.
- Two potential pathways for enhanced farmer uptake of multi-functionality:
  - Increase trust between farmers and actors that are currently agents of multi-functional messages;
  - Increase the multi-functional breadth of messages (mandate) of actors that are already trusted by farmers.

## Supplementary information

Supplementary Information
