# Supplementary material for: Trust Versus Content in Multi-functional Land Management: Assessing Soil Function Messaging in Agricultural Networks
Source: Environ Manage. 2022 Apr 22;69(6):1167–85. doi: 10.1007/s00267-022-01647-2 (PMC9079025; doi:10.1007/s00267-022-01647-2)
Supplement: Supplementary file 1 — Supplementary Information [file 267_2022_1647_MOESM1_ESM.docx]

S1. Supplementary Information

Latitude of acceptance


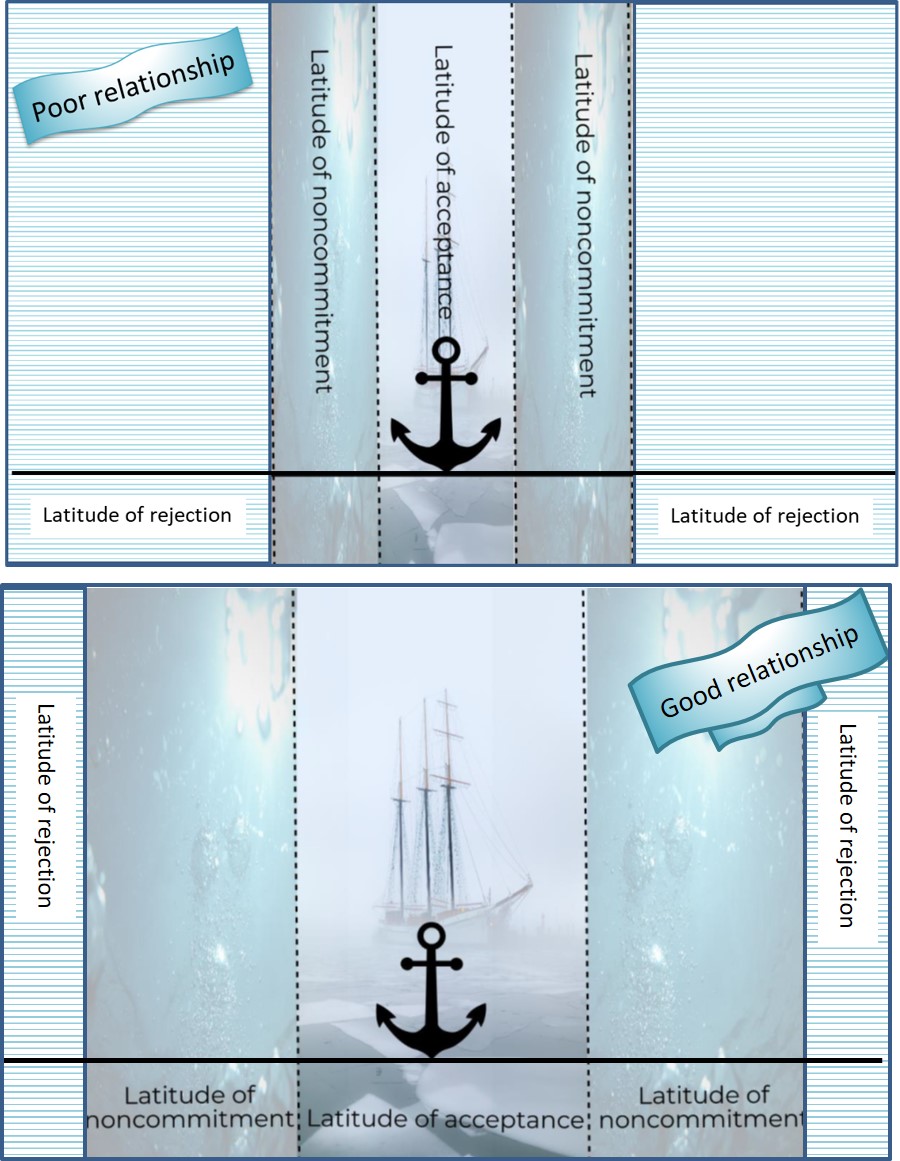


Figure S1 The role of the relationship in relation to the scale of latitudes as described in social judgement theory whereby higher levels of trust will increase the latitude of acceptance. Figures Adapted from de Vries et al. (2019).

Where a relationship is poor between actors will more likely result in messages being placed in the latitude of rejection. If an actor receives many messages that are in the latitude of rejection, it may even result in a shift of the anchor even further away from the messages. Where a good relationship exists, such messages are more likely to be in the zone of non-commitment or even acceptance.


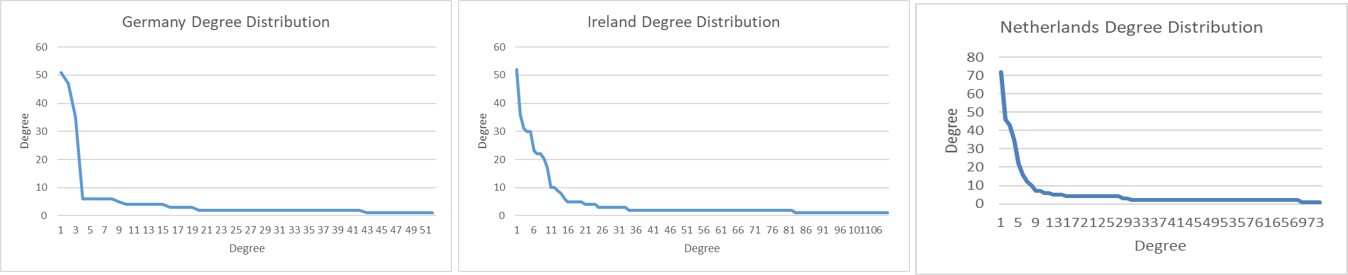


Figure S2 shows the degree distribution across the three case studies. The graphs reflect a ‘power law’ distribution. This indicates that there are a few nodes with a very high degree, with many more with a much lower degree.

T1 Full table listing of measures visualised in Figure 6.
